# Budget impact analysis of deep brain stimulation devices with different longevity in Parkinson's disease: insights from real-world data

**DOI:** 10.3389/fpubh.2026.1735033

**Published:** 2026-01-23

**Authors:** Mariachiara Sensi, Pietro Antenucci, Fabiana Colucci, Andrea Gozzi, Jay Guido Capone, Chiara Angelini, Fatima Waheed, Michele Alessandro Cavallo, Alba Scerrati

**Affiliations:** 1 Neurology Department, Arcispedale Sant'Anna Hospital, Ferrara, Italy; 2 Clinical Neurology Unit, Department of Neuroscience and Rehabilitation, University of Ferrara, Ferrara, Italy; 3 Parkinson and Movement Disorders Unit, Department of Clinical Neurosciences, Fondazione IRCCS, Istituto Neurologico Carlo Besta, Milan, Italy; 4 Neurosurgery Unit, Arcispedale Sant'Anna, Ferrara, Italy; 5 Department of Translational Medicine, University of Ferrara, Ferrara, Italy

**Keywords:** deep brain stimulation, economic evaluation, implantable pulse generator, Parkinson disease, real world evidence

## Abstract

**Background:**

Deep brain stimulation (DBS) is a viable treatment option for Parkinson's disease (PD) based on modulation of brain networks by electrical stimulation powered by implantable pulse generators (IPGs). Commercially available IPGs are either rechargeable (R) or non-rechargeable (NR). A budget impact analysis was developed to compare costs of the two options in PD-DBS patients, focusing on avoided battery replacements, associated complications, hospitalization rates.

**Methods:**

The study was conducted in two phases: an observational, single-center, retrospective analysis from 2005 to 2023 of 94 PD-DBS patients, followed by a second phase in which the economic data obtained were used to build a budget impact analysis (BIA) comparing the two different options.

**Results:**

Data from 47 PD-DBS patients with NR and 47 with R-IPGs were examined. BIA was calculated on a single patient with a 15-year and 25-year R-IPG. The higher initial cost of R devices compared with NR devices is offset by the absence of device replacements. This results in an estimated savings of €38,333 per patient with a 15-year R-IPG and €71,584 with a 25-year IPG.

**Conclusions:**

BIA predicts that the cost savings with a long-lasting R-IPG up to 25 years is cost effective compared with NR, regardless of age and life expectancy.

## Introduction

Parkinson's Disease (PD) is rapidly increasing worldwide (1, 2) imposing a significant burden on patients' quality of life and creating substantial economic challenges for both individuals and healthcare systems. The prevalence of the disease has doubled in the last 25 years: estimates for 2019 indicate over 8.5 million people affected (3). In Italy a rate of 193 patients per 100,000 inhabitants, with an annual incidence of 23.1 cases per 100 thousand inhabitants, was documented in 2020 (2). As PD progresses, there is a notable shift in the management costs from the National Health System to the patient, leading to increased “out-of-pocket” expenses. These costs rise significantly in advanced stage of the disease, particularly when motor fluctuations emerge (4–8).

Deep brain stimulation (DBS) is a surgical procedure for the treatment of complicated phase of PD. It is a reversible, safe, and efficacious therapy for various movement disorders, achieved by modulating cerebral networks through high-frequency electrical stimulation delivered by implantable pulse generators (IPGs) (9, 10).

Given the growing economic constraints and the need for efficient management of patients with progressive diseases, healthcare systems, particularly within National Health Systems, have undergone restructuring. To ensure long-term sustainability, the adoption of cost-effective therapies that mitigate the socio-economic burden of these conditions is imperative. DBS has been demonstrated as a cost-effective treatment, as corroborated by recent systematic reviews (11–14).

Initially, DBS was implemented using non-rechargeable battery-powered IPGs (NR-IPGs). These devices, with a lifespan ranging from 2 to 6 years depending on stimulation parameters, battery type, target site and patient-specific clinical factors (15), require surgical replacement upon battery depletion. This necessitates additional invasive procedures, exposing patients to increased discomfort and heightened risks of complications such as infection, skin erosion, device malposition, and malfunction (16, 17). The introduction of rechargeable IPGs (R-IPGs) has significantly mitigated many of these risks (17). R-IPGs present numerous advantages, including a reduced incidence of clinical complications (18–20), fewer replacement surgeries, and decreased hospital admissions. Moreover, R-IPGs have been associated with potential cost savings when compared to NR-IPGs (21, 22).

To date, the majority of economic evaluations have focused on the cost-effectiveness of DBS combined with best medical therapy vs. best or standard medical therapy alone (23, 24). However, only a limited number of studies have investigated the potential economic benefits of rechargeable devices specifically (14, 25–27).

Currently, several R-IPGs are available on the market from different manufacturers (Medtronic, Dublin, IR; Boston Scientific, Marlborough, MA, USA; Abbott Neuromodulation Lake County, IL; USA), with device longevity ranging up to 25 years (Vercise Genus R16, Boston Scientific) (28). The sole economic analysis in the literature pertains to R-IPGs with a lifespan of 9 years (25), and there is a lack of data regarding the budgetary impact of devices with the longest battery life, considering time horizons already well established in clinical practice (15 years) and the lifespan of newer models with maximum longevity (25 years).

## Objectives

The objective of this study is to develop a budget impact analysis (BIA) comparing the costs of R-IPGs vs. NR-IPGs in DBS for PD, focusing on real-world data concerning battery replacements, related complications and hospitalization rates.

## Methodology

### Study design

The study was conducted in two distinct phases: an observational, retrospective, single-center study, followed by the development of a BIA. The observational study analyzes real-life data from PD-DBS patients at the Sant'Anna Hospital in Ferrara (Italy) from 2005 to 2023 (18 years). In the second economic phase, data from the observational analysis jointly with costs were extrapolated for a time horizon of 25 years to build a BIA (29).

### Recruitment

Patients have been enrolled based on following inclusion criteria: PD patients age >18 years old, treated with Subthalamic Nucleus DBS following the literature selection criteria (30) implanted with dual channel IPG continuous stimulation. Patients with not complete data in medical records were excluded. The study was approved by the local ethics committee (489/2023/Oss/AOUFe).

### Data collection

Socio-demographic data, including age, sex, age at intervention, PD severity, disease duration, model of IPG implanted and stimulation parameters were collected. Resource utilization data covered the operative phase (operating room hours, neurosurgery staff time, DBS equipment, hospital stay), and any subsequent procedures for lead or IPG replacement and patient follow-up. Adverse events related to the procedure, medications (such as antibiotics used as prophylaxis of IPG replacements) or secondary to stimulation were documented in each phase.

Data were recorded at the time of implantation surgery and during each battery replacement. Battery lifespan was also tracked to estimate the number of replacements required over the study's time horizon. For patients who transitioned from NR-IPG to R-IPG, data reflect the pre-switch period.

Clinical and battery-related data were obtained from the hospital's electronic medical records, while resource utilization data came from hospital records. Cost data were sourced from literature and other publicly available references (31, 32). General cost estimates were used for complications. All data were anonymized, adhering to the ethical principles of the Helsinki Declaration and good clinical practice standards.

### Statistical analysis

A descriptive analysis of the database was first conducted. For continuous variables, mean and standard deviation were provided, while for categorical variables, frequencies were reported. Mean, standard deviation, and absolute and relative frequencies were calculated for qualitative variables.

To identify statistically significant differences between the two groups (R-IPGs and NR-IPGs), mean comparison tests were performed using the T-test or non-parametric alternatives (Mann-Whitney), based on the assumptions and validity of each test. For qualitative variables, the Chi-square or Fisher's test was employed.

The results from this initial analysis phase were used as inputs for the BIA developed in the second phase. The model estimates cost differences between the two battery types by simulating patient progression over time and calculating costs for the initial implant and each battery replacement, including device costs, complications, hospital days, and programming visits.

Several assumptions were made: first, the patient cohort was modeled on a single patient to estimate the economic impact of a strategy change and ensure results could be easily extrapolated to other cases in any Italian hospital. A global time horizon of 25 years, aligned with the longest lifespan of available rechargeable batteries (Boston Scientific) (28), was chosen. Furthermore, results were also reported for different time horizons (5, 10, 15, 20 and up to 25), allowing consultation for different time spans plausibly encountered in clinical practice.

For battery replacements, retrospective data from the NR-IPG group up to the fourth replacement were used. Beyond this point, we assumed the same input would apply for any further replacements.

In the R-IPG group, no replacements were observed in our retrospective data during the follow-up, as it was shorter than the model's time horizon. Therefore, the models were built assuming a single replacement after 15 years, as for most commercially available R-IPGs, or a single replacement after 25 years for those R-IPGs with a declared longevity of 25 years, and the same use of resources of NR-IPG replacement procedure were used for calculations.

To assess the robustness of the results, two further scenario analysis were conducted. First, identical data inputs were applied for the initial implantation (assuming the same length of hospital stay, and same complications rate) for both the rechargeable and non-rechargeable options, except different initial costs of the two devices. In the second scenario, the replacement time was assumed to be of 5 years similar to longevity reported by the manufactures for non-rechargeable devices.

The analysis was conducted from the perspective of the Italian public payer, specifically the national health system (Italy/Emilia-Romagna) (32).

## Results

### Descriptive clinical analysis

Ninety four patients met the inclusion criteria: 47 PD-DBS patients were in the NR-IPG group and 47 in the R-IPG for a total of 94 IPG implanted. Socio-demographic and disease characteristics are reported in Table 1.

**Table 1:** Characteristics of the population at baseline before DBS implant.

**Table 1: Population**	**NR (*n* = 47)**	**R (*n* = 47)**	***p*-value**
Age at intervention (years)	63.2 (7.4)	55.6 (7.1)	< 0.001
Sex			0.034
*Female*	23.0 (48.9%)	13.0 (27.7%)	
*Male*	24.0 (51.1%)	34.0 (72.3%)	
Years of illness at DBS treatment (years)	12.4 (4.7)	11.9 (4.0)	0.558
MDS-UPDRS III	22.5 (8.1)	23.0 (9.1)	0.696
MDS-UPDRS IV	6.3 (4.0)	5.2 (2.6)	0.857
Hoehn and Yahr	2.4 (0.5)	2.6 (0.4)	0.008

Data are presented as mean ± standard deviation or numbers and percentage, NR, carriers of non-rechargeable-IPG; R, carriers of rechargeable IPG; DBS, deep brain stimulation; MDS-UPDRS, movement disorder society unified Parkinson's disease rating scale.

Most of NR-IPG (68 %) were implanted in the first period of our experience with DBS (from 2005 to 2012) and 32% of them and all 47 R-IPG, in the period from 2012 to present.

Disease duration, disease severity (MDS UPDRS III and IV and H&Y stage) was similar among the two groups, while an older population in the NR group compared to R group (63.2 vs. 55.6 years) was documented.

Data about first DBS implantation and IPG model implanted are reported in Table 2. Peri-procedural complications (occurred during first implant hospitalization) were higher in the NR group (19.1%) in comparison to the R group (14.9%) although not significant. None of the early and late post-first implant complications led to additional IPG replacements. Days of hospitalization are significantly shorter in the R group (8.4 ± 2.5) respect to NR group (12.0 ± 3.9; *p* < 0.001).

**Table 2:** Characteristics of population at first implantation of DBS.

**Table 2: DBS first implantation**	**NR (*n* = 47)**	**R (*n* = 47)**	***p* value**
**Type of IPG**			
*Medtronic–Kinetra*	30 (63.8%)	-	
*Medtronic–Activa PC*	4 (8.5%)	-	
*Abbott–Libra XP*	4 (8.5%)	-	
*Boston–Vercise PC*	2 (4.3%)	-	
*Abbott–Infinity*	7 (14.9%)	-	
*Medtronic–Activa RC*	-	3 (6.3%)	
*Abbott–Brio*	-	2 (4.3%)	
*Boston–Vercise Genus*	-	8 (17.0%)	
*Boston–Vercise Gevia*	-	34 (72.4%)	
**Mean stimulation parameters**			
*Mean frequency (Hz)*	130.2 ± 27.2	129.1 ± 19.8	0.764
*Mean amplitude (mA)*	2.8 ± 0.7	2.7 ± 0.7	0.119
*Mean pulse width (usec)*	61.7 ± 10.2	61.4 ± 14.7	0.889
**Stimulation mode**			
*Monpolar ring mode*	38 (80.8%)	18 (38.3%)	< 0.001
*Double monopolar ring mode*	3 (6.4%)	2 (4.3%)	
*Bipolar*	2 (4.3%)	4 (8.5%)	
*Directional*	3 (6.4%)	21 (44.6%)	
*Mixed*	1 (2.1%)	2 (4.3%)	
Length of Stay for implant (days)	12.0 ± 3.9	8.4 ± 2.5	< 0.001

Mean frequency, mean amplitude, mean stimulation parameters are reported after one year of stimulation. Data are presented as mean ± standard deviation or numbers and percentage. NR, carriers of non-rechargeable-IPG; R, carriers of rechargeable-IPG; DBS, deep brain stimulation, IPG, implantable pulse generator; Hz, hertz; mA, milliampere; usec, microseconds.

In Table 3 data on subsequent NR-IPGs replacements are reported. IPG exchange was routinely planned, in order to prevent an unwanted reemergence of symptoms, when the IPGs reached the elective replacement indicator (ERI) status. All the IPG were replaced in a range of time of 2–4 months before complete exhaustion. In the NR group, we documented up to four device replacements following ERI (on average, every 3.8 years ± 1.0) for a total value of 60 replacements in the follow-up evaluation. In R group no IPGs replacements were documented during the observation period.

**Table 3:** Insights on non-rechargeable implant pulse generator battery replacements.

**Table 3: insights on battery replacements**	**NR (*n* = 47)**
1st IPG replacement (n)	36.0
ERI (%)	32.0 (88.9%^†^)
Early replacement due to IPG infection (%)	3.0 (8.3%^†^)
Early replacement due to unexpected EOS (%)	1.0 (2.8%^†^)
*Lead repositioning (%)*	2.0 (5.6%)
*Peri-procedural complications (%)*	4.0 (11.1%)
Delirium	3.0 (8.3%)
Sepsis	1.0 (2.8%)
*Operating room time (min)*	83.2 (26.5)
*Neurosurgeon time (min)*	43.8 (16.4)
*Length of stay (days)*	2.5 (1.5)
2nd IPG replacement (n)	26.0
ERI (%)	23.0 (88.5%^†^)
Early replacement due to IPG infection (%)	2.0 (7.7%^†^)
Early replacement due to unexpected EOS (%)	1.0 (3.9%^†^)
*Peri-procedural complications (%)*	1.0 (3.9%)
*Allergic reaction to prophylaxis (%)*	1.0 (3.9%)
*Operating room time (min)*	110.1 (15.4)
*Neurosurgeon time (min)*	50.4 (7.7)
*Length of stay (days)*	3.1 (3.7)
3rd IPG replacement (n)	6.0
ERI (%)	4.0 (66.7%^†^)
Early replacement due to IPG infection (%)	2.0 (33.3%^†^)
*Peri-procedural complications (%)*	-
*Neurosurgeon time (min)*	59.2 (4.9)
*Operating room time (min)*	81.7 (4.9)
*Length of stay (days)*	6.0 (10.1)
4th and following IPG replacements	1.0
ERI (%)	1.0 (100.0%^†^)
*Peri-procedural complications (%)*	-
*Operating room time (min)*	95.0 (0)
*Neurosurgeon time (min)*	47.0 (0)
*Length of stay (days)*	2.0 (0)
Total amount of days of admissions (days)	13.6
Mean battery duration (years)	3.8 (1.0)

^†^Percentage per total number of corresponding IPG replacements.

Total amount of days of admission was modeled on a single patient based on data from entire cohort. Data are presented as mean ± standard deviation or numbers and percentage. NR, carriers of non-rechargeable-IPG; R, carriers of rechargeable; IPG, implantable pulse generator; DBS, deep brain stimulation; IPG, implantable pulse generator; ERI, elective replacement status; EOS, end of service.

Secondary causes of replacement were IPG infection (7/47 patients) and sudden battery failure [Unexpected End of Service (EOS); 2/47]. All IPG infections except one occurred within a period of 28–50 months, following previous replacement. One IPG infection occurred within 3 months of the preceding replacement, which was performed regularly due to ERI. Additionally, two cases of EOS occurred 12 days and 34 months after a replacement of the previous IPG. No other adverse events were caused directly by IPGs replacement (like erroneous connection of the extension cable to the IPG and wound dehiscence).

One case of IPG infection reported in the R-IPGs group needed a surgical debridement without IPG replacement. Three NR-IPG carriers switched to R-IPGs after first replacement for ERI after a mean of 44.3 (± 4.9) months. All of them chose to switch because of discomfort for subsequent multiple surgical procedures. None of our 47 R patients asked to switch to NR for discomfort in recharging procedure.

Considering two standard days of hospitalization needed in our hospital organization for each replacement, the NR group accumulated a total of 120 hospitalization days for programmed ERI replacements and 64 hospitalization days due to IPG-related complications that required early or unscheduled replacement. This corresponds to 0.4 additional days for patient for each first scheduled replacement, 0.9 days for each second replacement, and 4.7 days for each third replacement. Finally, considering all hospitalization days, including the standard time for scheduled replacements and days related to IPG complications, the average hospitalization time for a patient with an NR-IPG, as estimated from the cohort up to the fourth replacement, is 13.6 days. No days of hospitalization were documented in RC group after the first implant due to subsequent replacements.

Table 4 shows the resulting costs after a micro costing process that estimates the costs of the DBS procedure, which includes the DBS device, follow-up visits for device programming, IPG, Leads, and procedural costs (staff, tests, operating room and materials, ECG, and standard preoperative blood tests), or IPG replacements. Associated costs of antibiotics, either preoperatory with cefazolin or vancomycin before the surgical incision, or during complications with amoxicillin and clavulanic acid, and ceftriaxone are not included as these are very low and neglectable for budget impact calculations.

**Table 4:** Cost of the procedure from the national/regional health system (Italia/Emilia-Romagna Sistema Sanitario Nazionale) payer perspective.

**Table 4: costs**	**Value (€)**
**DBS treatment**	
*DBSprocedure* ^†^	18.100 € (NR); 25.400 € (R)
*Complications (cost per complication)*	661 €
*Length of stay (cost per bed day)*	676 €
*Follow-up visits for programming optimization (cost per visit)*	23 €
**Replacements**	
*IPG replacement (cost per IPG & procedural costs)*	15.700 € (NR); 23.000 € (R)
*Lead repositioning procedures (cost per LEAD & procedural costs)*	2.600 €
*Complications (cost per complication)*	661 €
*Length of stay (cost per bed day)*	676 €

^†^Includes DBS device, IPG, leads, procedural costs (staff, tests, operating room and materials) Costs are expressed in euros (€) and refer to the year 2023. DBS, deep brain stimulation; IPG, implantable pulse generator; NR, non-rechargeable; R, rechargeable.

### Budget impact analysis

In the BIA, we present the effect of treating a patient with one strategy vs. the other over 25-year time horizon.

**Figure 1:**
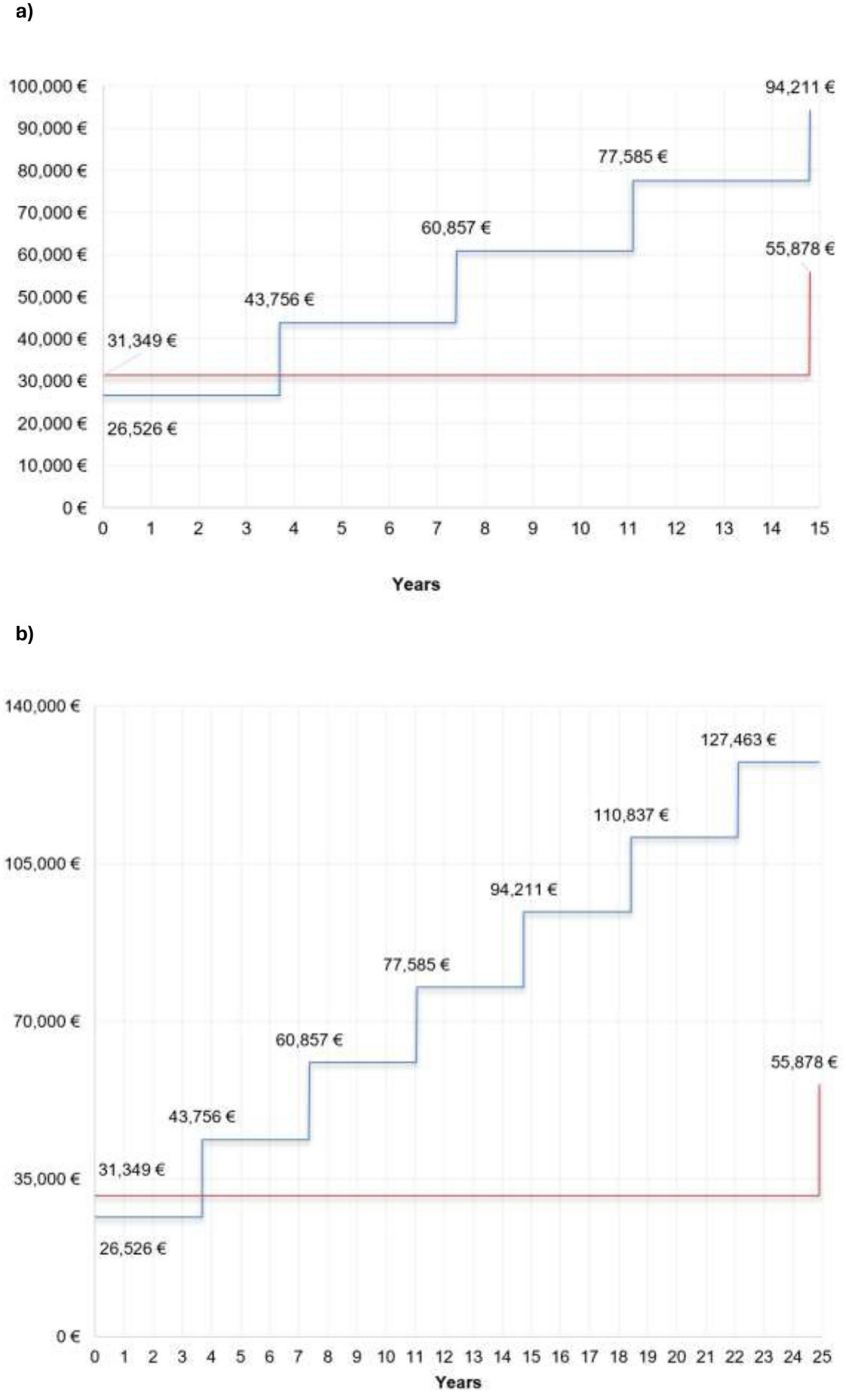
**(a)** Fifteen-year cost estimation (replacement after 15 years for the rechargeable implantable pulse generator). Blue line, Non-rechargeable, Red line, Rechargeable. (**b**) Twenty-five-year cost estimation (replacement after 25 years for the Rechargeable Implantable Pulse Generator). Blue line, Non-rechargeable, Red line, Rechargeable.

**Figure 2:**
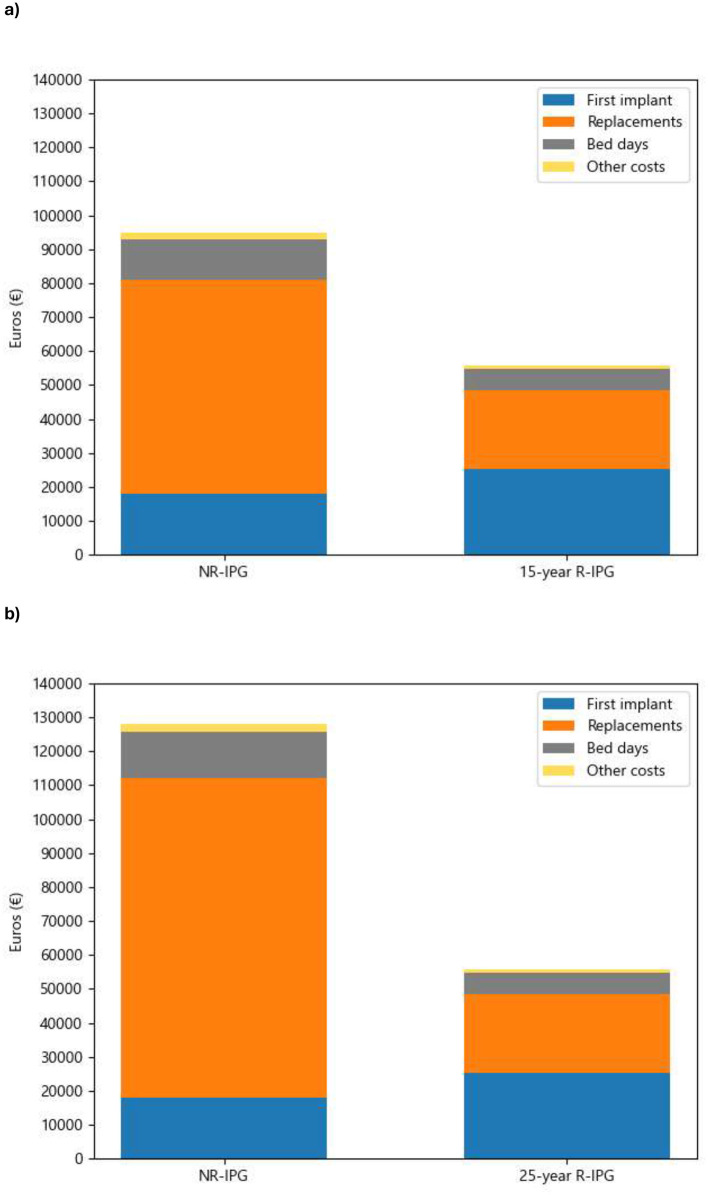
**(a)** Accumulated 15-year cost breakdown. NR, non rechargeable; R, rechargeable; IPG, implantable pulse generator. Other costs include programming visits and complications. (**b)** Accumulated 25-year cost breakdown. NR, non rechargeable; R, rechargeable; IPG, implantable pulse generator. Other costs include programming visits and complications.

Table 5 and Figures 1a and b presents cost savings for both considered time horizon, based on real-world data. The results favor the R-IPG, primarily due to avoided IPG replacements. The higher initial cost of the R-IPG is compensated by the absence of device replacements over the time horizon, resulting in estimated savings of €38.333 per patient considering a 15-year replacement in the rechargeable group and €71,585 per patient over 25 years. Figures 2a and b shows the accumulated cost breakdown for the same time horizon considered.

**Table 5:** Economic savings comparing rechargeable (R) vs. non-rechargeable implantable pulse generators (IPG).

**Time horizon (years)**	**Savings (–€)**	**Substitutions**	**Bed days**
1	4,822 €	0	−4
5	−12,406 €	−1	−6
10	−29,507 €	−2	−7
15 (with replacement of 15-year R-IPG)	−38,333€	−3	−8
15 (assuming a 25-year R-IPG)	−62,862 €	−4	−9
20	−79,488 €	−5	−10
25 (with replacement of 25-year R-IPG)	−71,585 €	−5	−9

Estimates are based on real-world data from the cohort under analysis.

Two additional scenarios were assessed. The first scenario (Table 6) assumed identical hospitalization costs for the first implant for both R-IPG and NR-IPG, except for the difference in device cost. Under this assumption, the model yielded cost savings of −38,556€ over 15 years and −69,108€ over 25 years. The second scenario (Table 7) assumed an IPG replacement interval of 5 years instead of the observed 3.8 years. This more conservative assumption resulted in cost savings of −21,707€ over 15 years and −54,959€ over 25 years, as fewer replacements would occur.

**Table 6:** First scenario, economic savings assuming identical implantation costs for the two devices, over different time horizons.

**Time horizon**	**Savings (–€)**	**Replacements**	**Bed days**
15 (15-year R-IPG)	−38,556 €	−3	−4
15 (25-year R-IPG)	−60,385 €	−4	−6
25 (25-year R-IPG)	−69,108 €	−5	−9

R-IPG, rechargeable–implantable pulse generator.

**Table 7:** Second scenario, economic savings assuming a 5-year replacement interval for non-rechargeable devices, over different time horizons.

**Time horizon**	**Savings (–€)**	**Replacements**	**Bed days**
15 (15-year R-IPG)	−21.707 €	−2	−4
15 (25-year R-IPG)	−46,236 €	−3	−6
25 (25-year R-IPG)	−54,959 €	−4	−8

R-IPG, rechargeable–implantable pulse generator.

These results demonstrate that, even when assuming equal hospitalization costs at the first implant and a longer replacement interval than the one observed in our analysis, the cost savings associated with the rechargeable device remain substantial at both time points.

## Discussion

Direct and indirect costs of PD are rising sharply in line with the exponential growth of patients and the increasingly limited financial resources of the national health systems.

A 2016 Italian study (33) reported that the average total expenditure per capita for PD patients aged over 50 was €4,441. In a prior study (6), the total costs of PD were calculated to be €8,640 per patient over a 6-month period, with direct costs accounting for 70% of the total. Indirect costs are also significant highlighting various estimates of annual productivity losses (34, 35).

Considering the costs' impact related to DBS procedure in PD patients, many studies previously had analyzed the cost of the first implant and/or the subsequent replacements (14, 25–27). Due to the high cost of these devices, it is crucial to appropriate handling of DBS equipment costs, and evaluate within the appropriate time horizon considering the eventuality of repeat DBS procedures, adverse effects, and economic impacts upon medication use affected by DBS. There are different suppliers on the market who produce this equipment, and the prices are quite similar for the first implant full equipment but is important to highlight that the cost could vary in subsequent replacements considering different IPGs longevity among NR and R ones and different prices imposed by different manufactures (36). Nowadays, according to the different manufacturers, an R-IPG replacement should be considered after 10 years (Brio, Abbott), 15 years (Percept RC, Medtronic), or 25 years (Boston Scientific) (28). For most models, these time references do not necessarily correspond to complete battery depletion, whereas in other cases (Activa RC) the indicated time closely reflects the ERI status.

Across All DBS studies IPG replacement account for about 9% of the total cost of DBS therapy and proportionally increases over the lifetime of the patient (12).

Rizzi et al. (14) firstly compared the implantation costs of NR-IPGs to the estimated costs of R-IPGs over a follow-up period of 7.9 years (with IPGs replacement of an average of 1.5 times per patient). Cost analysis revealed an overall cost saving of 234,194 euros, including the costs of management of complications. The cost calculation, considering Italian mean life expectancy (47 years), found that the expected expenditure with NR-IPGs would have been 17,669,942 euros, and for R-IPGs of 11,751,754 with a saving of 5,918,188 euros. The average annual savings expected for each patient would be 905 euros.

According to Hitti et al. (25) this cost-saving effect would be 60,900$ over the course of 9 years. In a study by Perez et al. (26) rechargeable devices are estimated to reduce costs by 34%.

Retrospective data show that patients with NR-IPGs were older, had longer hospital stays, and required more neurological evaluations after first implant than R-IPG patients. This likely reflects that most NR devices were implanted early in our experience (2000–2012), when inclusion criteria differed (37) and advanced programming features—directional leads, broader stimulation parameters, and software for reconstructing the volume of activated tissue—were unavailable. In recent years (2012–present), R devices are mainly used, with refined selection criteria favoring younger patients with shorter disease duration (38) and the latest technological advancements.

A key factor favoring R-IPGs was their lower infection risk due to fewer replacements. DBS infections incur significant costs from hardware salvage, explantation, and replantation (39). Reported infection rates range from 0.7% to 6% for NR-IPGs (40, 41) vs. 2% for primary R-IPG implantation (21, 22). Our long-term data align, showing 6.03% for NR-IPGs and 2.12% for R-IPGs.

Patient preference is another key factor in choosing between the two devices (42). While neurologists worry that older patients may struggle with R-IPG recharging due to cognitive decline, technology unfamiliarity, or limited caregiver support, recent surveys show most manage it well, with few adverse events or therapy interruptions (25, 43). Our long-term R-IPG experience confirms this: 10/47 reported minor recharging issues, no sudden IPG depletion occurred, and 8/47 needed caregiver help. Additionally, 3/47 NR patients switched to R-IPGs despite advanced age (mean 72 years), long disease duration (mean 24 years), and cognitive challenges. Nevertheless, in the decision-making process, it should be kept in mind that if the patient or the healthcare facility responsible for their care is no longer able to manage regular recharging, elderly patients who still benefit from DBS therapy may need to consider switching to an NR-IPG.

The second part of our study involved a BIA comparing economic costs of R-IPG and NR-IPG batteries. The model shows the impact of using R-IPGs in our hospital. Figure 1 illustrates that every 3.8 years (based on NR-IPG replacement data), NR-IPG costs rise, while R-IPGs require only one replacement at battery exhaustion, modeled in two scenarios. Over time, R-IPGs become more cost-effective despite NR-IPGs offering short-term savings, including scenarios with equal initial hospitalization costs or hypothetical 5-year battery longevity.

These data are confirmed by Holm et al. (27) which, in a scenario modeled for a battery longevity calculated for 9 + 15 years (Extended RC Medtronic) found that R devices could reduce costs from 44 to 51%, in a 16-years horizon. Savings were greater with the 15-year compared with 9-year longevity IPGs. It is therefore likely that a R device with a longevity of 25 years could be even more economically advantageous than one with a longevity of 10 or 15 years.

Our model also assessed the economic impact of shifting to R-IPGs on hospitalization costs. For a single patient with an R-IPG over 25 years, a hospital would avoid 5 IPG replacements (–€71,584 per patient). This strategy also enhances hospital operational capacity, particularly in contexts of high demand and long waiting lists.

Considering the projected economic burden of Parkinson's in the coming years (44–46), these long-term projections are especially valuable, given differences in reimbursement systems across countries and the rapidly evolving public health economic landscape (21, 22).

In Italy, as in much of Europe, all device-assisted therapies (DAT), such as apomorphine or levodopa infusions, are now fully reimbursed. While DBS has a higher initial cost for neurosurgery and the stimulator system compared to infusion therapies [with an annual cost of €40,000 to €80,000 (47)], long-term projections show that DBS is cost-saving compared to other DATs (45, 46).

DBS equipment costs may change over time for both R- and NR-IPGs. A typical PD-DBS patient (aged 50–65) with a 25-year life expectancy (LE) may need 4–5 NR-IPGs. For PD onset between 40 and 64 years [LE estimated around 21 years (48)], a 25-year R-IPG is conceptually more favorable than 10- or 15-year devices. Our data also support this choice for patients with onset after 65, as a French study reported an LE of 16 years (17.8 for women, 16.1 for men) (49). In both age groups, our budget analysis shows a 25-year R-IPG is consistently less costly than 15-year R-IPGs

Compared to previous studies (14, 25–27), our study has several strengths and limitations. Its single-center design ensures uniform surgical techniques and postoperative DBS management but may limit generalizability. Major strengths include the large sample size, long-term follow-up, and budget calculations based on real-life data. Limitations include its retrospective nature, lack of detailed complication costs, and extrapolation of 18-year follow-up data to a 25-year economic horizon. Economic data reflect the longevity of the most commonly used device models, which may change in the next future. Extensive clinical experience exists for R-IPGs with up to 15-year battery life, whereas devices rated for 25 years are newer and less evaluated; actual lifespan may be shorter due to complications and other factors (14, 16). Developed from Italian clinical practice, findings may vary elsewhere, but are likely applicable across Europe, considering different device lifespans.

## Conclusions

This study confirms that the economic savings of using a R system are evident in the short term and grow exponentially at both analyzed time points. This provides an important indication, together with increasingly emerging data (43, 50) of the agreeability of patients and caregivers, to prefer R devices in all PD-DBS patients regardless of age, disease duration and life expectation taking in account future costs related to disease progression.

## Data Availability

The raw data supporting the conclusions of this article will be made available by the authors, without undue reservation.
